# Assessment of the renal angina index in patients hospitalized in a cardiac intensive care unit

**DOI:** 10.1038/s41598-023-51086-0

**Published:** 2024-01-02

**Authors:** Eirin Sakaguchi, Hiroyuki Naruse, Yuya Ishihara, Hidekazu Hattori, Akira Yamada, Hideki Kawai, Takashi Muramatsu, Yoshiki Tsuboi, Ryosuke Fujii, Koji Suzuki, Junnichi Ishii, Kuniaki Saito, Masayoshi Sarai, Masanobu Yanase, Yukio Ozaki, Hideo Izawa

**Affiliations:** 1 Department of Faculty of Medical Technology, Fujita Health University School of Medicine, 1-98 Dengakugakubo, Kutsukake-cho, Toyoake, Aichi 470-1192 Japan; 2 Department of Cardiology, Fujita Health University School of Medicine, 1-98 Dengakugakubo, Kutsukake-cho, Toyoake, Aichi 470-1192 Japan; 3 Department of Preventive Medical Sciences, Fujita Health University School of Medicine, 1-98 Dengakugakubo, Kutsukake-cho, Toyoake, Aichi 470-1192 Japan; 4 Department of Cardiology, Fujita Health University School of Medicine, Okazaki Medical Center, 1 Aza Gotanda, Harisaki-cho, Okazaki, Aichi 444-0827 Japan

**Keywords:** Biomarkers, Cardiology, Medical research, Nephrology

## Abstract

The renal angina index (RAI) is a validated scoring tool for predicting acute kidney injury (AKI). We investigated the efficacy of the RAI in 2436 heterogeneous patients (mean age, 70 years) treated in cardiac intensive care units (CICUs). The RAI was calculated from creatinine and patient condition scores. AKI was diagnosed by the Kidney Disease: Improving Global Outcome criteria. The primary and secondary endpoints were the development of severe AKI and all-cause mortality, respectively. Four hundred thirty-three patients developed AKI, 87 of them severe. In multivariate analyses, the RAI was a significant independent predictor of severe AKI. During the 12-month follow-up period, 210 patients suffered all-cause death. Elevated RAI was independently associated with all-cause mortality, as was NT-proBNP (*p* < 0.001). The RAI is a potent predictor not only of severe AKI but also of adverse outcomes and substantially improved the 12-month risk stratification of patients hospitalized in CICUs.

## Introduction

The renal angina index (RAI), designed to reflect small changes in serum creatinine levels and patient condition, is a validated scoring tool for predicting severe acute kidney injury (AKI) in critically ill children and adolescents^1,2^. The RAI has not been used in adult patients because it was derived in the pediatric population, which differs significantly from adult patients with respect to comorbidities and risk factors for AKI. Recent studies have demonstrated the performance of the RAI using a concise scoring system in adult patients in intensive care units^3^, but the usefulness of the RAI in patients with cardiovascular disease remains unclear.

AKI is a detrimental syndrome that is common in patients admitted to cardiac intensive care units (CICUs)^4–6^. Owing to its high morbidity and mortality^7,8^, AKI is associated with longer hospital stays, higher resource utilization, and higher overall healthcare costs^9^.

In the present study, we investigated the predictive value of the RAI for AKI and the prognostic impact of the RAI in adult patients hospitalized in CICUs.

## Materials and methods

### Study design

This study was conducted in the Department of Cardiology, Fujita Health University School of Medicine (Toyoake, Japan). The Ethics Committee of Fujita Health University approved this study (study protocol number: HM19-264), which was conducted in accordance with the Declaration of Helsinki. Written informed consent was obtained from all the patients.

Patients hospitalized in CICUs between November 2009 and December 2018 were enrolled in this study. Patients who had the following characteristics were excluded from participation: (1) age under 18 years; (2) absence of serum creatinine (SCr), N-terminal pro-B-type natriuretic peptide (NT-proBNP), or left ventricular ejection fraction (LVEF) data; (3) stage 5 chronic kidney disease (CKD); (4) stay in the CICU < 24 h; and (5) history of kidney transplantation. Physicians independently selected the appropriate therapy and managed the patients following standard protocols using outcome measurements as feedback, such as improvement in symptoms, physical examination findings, laboratory data, pulmonary congestion on chest radiography, and echocardiographic findings. Clinical characteristics were obtained from the patients’ medical records upon enrollment.

### Definitions and calculations

AKI was diagnosed under the Kidney Disease: Improving Global Outcomes (KDIGO) criteria, as an increase in SCr by ≥ 0.3 mg/dL within 48 h or an increase in SCr to ≥ 1.5 times the baseline within 1 week^10^. Severe AKI was defined as stage 2 or 3 AKI according to the KDIGO criteria^10^. KDIGO characterizes stage 2 AKI as a greater than or equal to twofold increase in baseline SCr within 7 days and stage 3 AKI as a greater than or equal to threefold increase in baseline SCr within 7 days, an increase in SCr to greater than or equal to 4 mg/dL within 48 h, or the initiation of renal replacement therapy (RRT). Urinary criteria were not used to diagnose AKI because of inconsistent data and potential alterations in urine volume induced by medical therapy. The primary endpoint was the development of severe AKI.

The SCr-based estimated glomerular filtration rate (eGFR) was calculated using the CKD-EPI equations^11^. Incident end-stage kidney disease (ESKD) indicates the initiation of maintenance dialysis therapy, receipt of RRT during hospital stay, or kidney transplantation. CKD was defined as an eGFR of < 60 mL/min/1.73 m^2^. We routinely performed two-dimensional echocardiography to calculate LVEF using the modified Simpson method.

### Renal Angina Index

**Figure 1:**
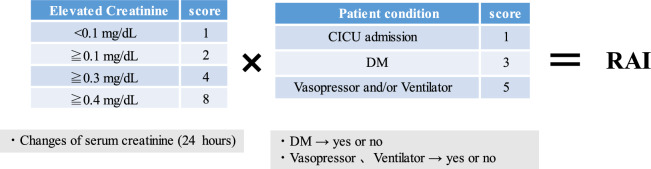
The elements of the RAI. The RAI score was defined as the creatinine score multiplied by the worst patient condition score. *CICUs* cardiac intensive care units; *DM* diabetes mellitus; *RAI* renal angina index.

The previously reported RAI^3^ was used for patients hospitalized in CICUs (Fig. 1). The RAI was calculated from creatinine and patient condition scores. The creatinine scores were assigned according to the changes in SCr within 24 h after CICU admission as follows: SCr ≥ 0.4 mg/dL, 8 points; SCr ≥ 0.3 mg/dL, 4 points; SCr ≥ 0.1 mg/dL, 2 points; and SCr < 0.1 mg/dL, 1 point. The condition of each patient was scored as follows: ventilation and/or vasopressor therapy, 5 points; diabetes mellitus, 3 points; and admission to the CICUs, 1 point. The RAI equaled the creatinine score multiplied by the worst patient condition score. Its possible values were 1, 2, 3, 4, 5, 6, 8, 10, 12, 20, 24, and 40.

### Outcomes

All patients were clinically followed up for 12 months after study enrollment. The secondary endpoint, judged independently by the researchers, was all-cause mortality. Endpoint data were obtained from hospital charts and telephone interviews with patients. Telephone interviews were conducted by trained reviewers blinded to the study details.

### Measurement of biochemical markers

Serum NT-proBNP was measured using an electrochemiluminescence immunoassay with the Cobas e601 system (Roche Diagnostics, Tokyo, Japan). SCr concentration was determined by an enzymatic method using the Liquitech® Creatinine PAP II (Roche Diagnostics, Tokyo, Japan) on admission, daily through day three, and then on day seven.

### Statistical methods

JMP version Pro 15 software (SAS Institute Inc., Cary, NC, USA) and R Version 4.2.1 (R Foundation for Statistical Computing, Vienna, Austria) were used for statistical analyses. Data are presented as number and frequency for categorical variables and mean ± standard deviation or median with interquartile ranges for continuous variables.

Clinical characteristics were compared using the chi-squared test for categorical variables and the Mann–Whitney U test and Student’s t test for continuous variables. The odds ratios and 95% confidence intervals (CIs) were calculated for each factor using logistic regression, and all baseline variables (*p* < 0.05) in the univariate analyses were entered into the multivariate model to determine the independent predictors of severe AKI.

All baseline variables with *p* < 0.05 in the univariate analyses were integrated into the Cox multivariate model to determine the independent predictors of all-cause mortality. Hazard ratios and 95% CIs were calculated for each factor using Cox proportional hazards analysis. Receiver operating characteristic (ROC) curves were drawn to assess the ability of the RAI to differentiate between patients with and without severe AKI. In ROC analyses, the optimal cutoff value was defined as the level with the largest sum of sensitivity and specificity. We used R software to calculate the area under the ROC curve (AUC) and CIs by the bootstrapping method with 2,000 iterations. To compare different ROC curves from multi variable models without or with RAI, we used a function of roc.test() in the pROC package^12^. Kaplan–Meier curves were plotted and compared using the log-rank test. Statistical significance was set at *p* < 0.05.

## Results

Of the 6,227 consecutive patients hospitalized in CICUs, 3,774 were excluded for the following reasons (younger than 18 years in 16 patients; absence of SCr, NT-proBNP, or LVEF data in 3,364 patients; stage 5 CKD in 377 patients; length of stay in CICUs < 24 h in 32 patients; and kidney transplantation in 2 patients). Thus, 2,436 patients (1,572 males; mean age, 70 years) were enrolled in this study (Table 1). A total of 1,083 patients (44%) were admitted because of acute coronary syndrome (386 patients had ST-segment elevation myocardial infarction, 555 had non-ST-segment elevation myocardial infarction, and 142 had unstable angina); 871 (36%) because of acute decompensated heart failure (469 patients with reduced ejection fraction [LVEF < 40%], 132 with mid-range ejection fraction [40% ≤ LVEF < 50%], and 270 with preserved ejection fraction [LVEF ≥ 50%]); 167 (7%) because of arrhythmia; 74 (3%) because of acute aortic syndrome; 59 (2%) because of pulmonary embolism; 30 (1%) because of infective endocarditis; and 152 (6%) due to other causes.

**Table 1:** Baseline characteristics according to all-cause death.

	All	Survivors	Nonsurvivors	*p* value
Number	2,436	2,226	210	
Male	1,572 (65)	1,438 (65)	134 (64)	0.82
Age (years)	70.4 ± 13.4	69.9 ± 13.5	75.2 ± 10.6	< 0.001
Hypertension	1,555 (64)	1,425 (64)	130 (62)	0.54
Diabetes mellitus	861 (35)	784 (35)	77 (37)	0.68
Hyperlipidemia	1,049 (43)	974 (44)	75 (36)	0.02
Hyperuricemia	569 (23)	504 (23)	65 (31)	0.01
Previous myocardial infarction	366 (15)	315 (14)	51 (24)	< 0.001
Chronic kidney disease	1,108 (45)	959 (43)	149 (71)	< 0.001
Acute coronary syndrome	1,083 (44)	1,029 (46)	54 (26)	< 0.001
Acute decompensated heart failure	871 (36)	754 (34)	117 (56)	< 0.001
Systolic blood pressure (mmHg)	137 ± 27	137 ± 27	133 ± 26	0.03
Heart rate (bpm)	88 ± 30	88 ± 30	92 ± 26	0.08
Hemoglobin (g/dL)	12.4 ± 2.2	12.5 ± 2.2	11.3 ± 2.3	< 0.001
Serum creatinine at admission (mg/dL)	0.97 ± 0.44	0.94 ± 0.42	1.24 ± 0.60	< 0.001
Serum creatinine after 24 h (mg/dL)	1.03 ± 0.51	0.99 ± 0.48	1.36 ± 0.70	< 0.001
eGFR at admission (mL/min/1.73 m^2^)	80.9 ± 24.9	82.4 ± 24.3	64.7 ± 25.6	< 0.001
NT-proBNP (pg/mL)	1501 (290–4,907)	1250 (259–4,364)	6131 (2,646–16,632)	< 0.001
Troponin I (ng/mL)	0.17 (0.03–2.55)	0.16 (0.03–2.39)	0.30 (0.05–3.78)	0.94
Ventilation at enrollment	459 (19)	378 (17)	81 (39)	< 0.001
Vasopressor at enrollment	483 (20)	386 (17)	97 (46)	< 0.001
IABP or PCPS at enrollment	174 (7)	154 (7)	20 (10)	0.18
CAG or PCI before admission	792 (33)	746 (34)	46 (22)	< 0.001
RAI	3 (1–5)	3 (1–5)	5 (3–10)	< 0.001
LVEF (%)	47 ± 14	47 ± 14	39 ± 15	< 0.001
Treatment at discharge				
ARBs or ACE inhibitors	1,389 (57)	1,325 (60)	64 (30)	< 0.001
Beta-blocker	1,399 (57)	1,303 (59)	96 (46)	< 0.001
MRA	645 (26)	577 (26)	68 (32)	0.05

Data are presented as number (%), mean ± standard deviation, or median (interquartile range). *bpm* beats per minute; *eGFR* creatinine-based estimated glomerular filtration rate; *NT-proBNP* N-terminal pro-B-type natriuretic peptide; *IABP* intra-aortic balloon pumping; *PCPS* percutaneous cardio pulmonary support; *CAG* coronary angiography; *PCI* percutaneous coronary intervention; *RAI* renal angina index; *LVEF* left ventricular ejection fraction; *ARB* angiotensin receptor blocker; *ACE* angiotensin-converting enzyme; *MRA* mineralocorticoid receptor antagonist.

**Figure 2:**
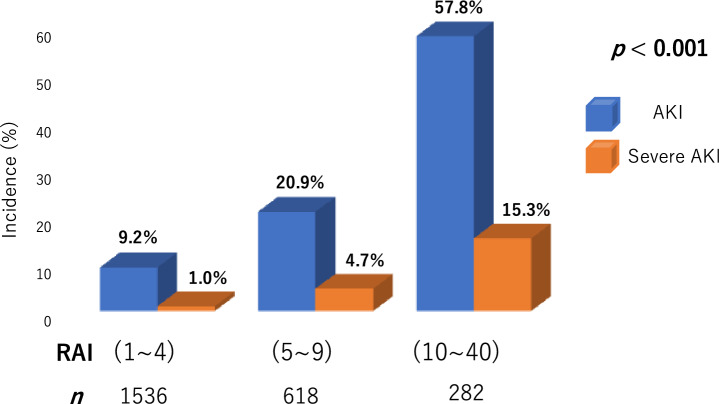
Incidences of AKI and severe AKI according to the RAI values. *AKI* acute kidney injury; *RAI* renal angina index.

**Figure 3:**
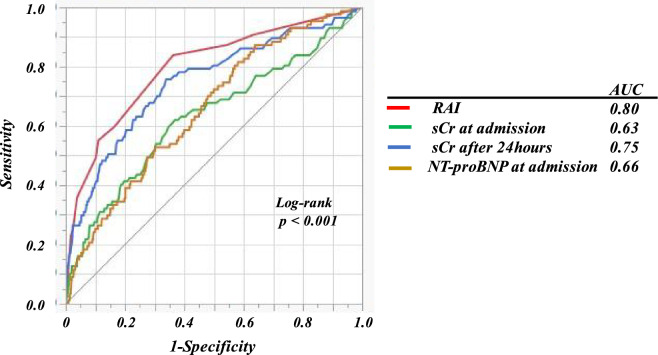
Receiver operating characteristic curves of RAI and creatinine at admission and after 24 h as well as NT-proBNP for predicting severe AKI. *AKI* acute kidney injury; *AUC* area under the receiver operating characteristic curve; *NT-proBNP* N-terminal pro-B-type natriuretic peptide; *RAI* renal angina index, *sCr* serum creatinine.

Of the 2,436 patients, 433 (18%) developed AKI, including 87 patients with severe AKI. Patients with severe AKI had higher creatinine on admission and after 24 h, NT-proBNP, troponin I, and RAI; and had lower hemoglobin, eGFR, and LVEF than those without (Supplementary Table S1). Many patients who developed severe AKI had the following characteristics: hypertension, CKD, acute decompensated heart failure, a history of mechanical ventilation and/or vasopressor use, and intra-aortic balloon pumping (IABP) or percutaneous cardio pulmonary support (PCPS) use. We divided the patients into three groups according to their RAI values. Patients with higher RAI values had a higher risk for severe AKI, as well as the risk of AKI (Fig. 2). As revealed in the multivariate logistic analysis that included all baseline variables with *p* < 0.05 by univariate analysis (Supplementary Table S2), the RAI was a significant independent predictor of severe AKI (Table 2). LVEF and IABP or PCPS were also significantly associated with severe AKI. In the ROC analyses, RAI differentiated with high precision the patients who developed severe AKI from those who did not (Fig. 3; *p* < 0.001). The AUC generated for RAI (0.80) was higher than that for SCr at admission and after 24 h and NT-proBNP (0.63, 0.75, and 066, respectively). The bootstrap-corrected AUC value for the multivariable model including RAI was significantly higher than that excluding RAI [0.83 (0.79–0.87) vs. 0.76 (0.71–0.81); *p* < 0.001]. The optimal cutoff value of the RAI for predicting severe AKI was 4 points (sensitivity, 0.84; specificity, 0.64).

**Table 2:** Multivariate logistic analyses of predictors for severe AKI.

Variables	OR (95% CI)	*p* value
Hypertension	1.50 (0.89–2.53)	0.12
Chronic kidney disease	1.75 (0.83–3.66)	0.14
Acute decompensated heart failure	1.32 (0.75–2.33)	0.34
Hemoglobin (per 1 SD increment)	0.79 (0.62–1.01)	0.06
eGFR on admission (per 1 SD increment)	1.24 (0.87–1.77)	0.23
Log NT-proBNP (per 1 SD increment)	1.33 (0.95–1.86)	0.10
LVEF (per 1 SD increment)	1.35 (1.04–1.76)	0.02
IABP or PCPS at enrollment	4.27 (2.23–8.18)	< 0.001
Log RAI (per 1 SD increment)	2.78 (2.19–3.53)	< 0.001

The multivariable model was adjusted for all baseline variables with *p* < 0.05 in univariate analyses. *OR* odds ratio; *CI* confidence interval; *SD* standard deviation; *eGFR* creatinine-based estimated glomerular filtration rate; *Log NT-proBNP* logarithm N-terminal pro-B-type natriuretic peptide; *LVEF* left ventricular ejection fraction; *IABP* intra-aortic balloon pumping; *PCPS* percutaneous cardio pulmonary support; *Log RAI* logarithm renal angina index.

During the 12-month follow-up period, 210 patients (8.6%) suffered all-cause death, 135 of whom had cardiovascular deaths (Table 1). Cardiovascular deaths were caused by heart failure in 89, myocardial infarction in 29, arrhythmia in 8, stroke in 4, sudden death in 3, and aortic dissection in 2 patients. ESKD was observed in 20 patients, 17 of whom were alive and 9 of whom were independent of dialysis at 12 months. Patients who died were older; had higher creatinine on admission and after 24 h, NT-proBNP, and RAI; and had lower systolic blood pressure, hemoglobin, eGFR, and LVEF than those who did not. Many patients who died had the following characteristics: hyperuricemia, previous myocardial infarction, CKD, acute decompensated heart failure, a history of mechanical ventilation and/or vasopressor use, and mineralocorticoid receptor antagonist (Table 1). Nonsurvivors less frequently had the following characteristics: hyperlipidemia, acute coronary syndrome, emergent coronary angiography or percutaneous coronary intervention before admission, angiotensin receptor blockers (ARBs) or angiotensin-converting enzyme (ACE) inhibitors, and beta-blockers.

**Figure 4:**
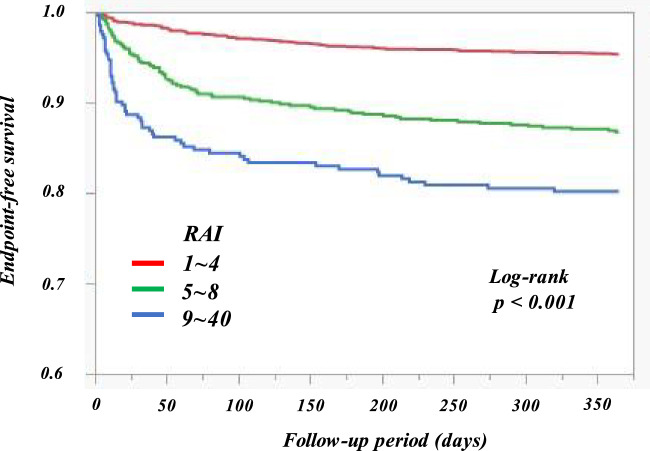
Kaplan‒Meier curves for 12-month mortality. *RAI* renal angina index.

As revealed in the Cox multivariate analysis that included all baseline variables with *p* < 0.05 by univariate analysis (Supplementary Table S3), the RAI was a significant independent predictor of all-cause mortality (Table 3). Previous myocardial infarction, hemoglobin, NT-proBNP level, and LVEF were also significantly associated with all-cause mortality. The bootstrap-corrected AUC value for the multivariable model including RAI was significantly higher than that excluding RAI [0.79 (0.76–0.82) vs. 0.78 (0.75–0.81); *p* = 0.04]. When patients were divided into three groups according to RAI value, the Kaplan–Meier curves revealed a graded increase in the risk of all-cause death with higher RAI (Fig. 4; *p* < 0.001).

**Table 3:** Multivariate cox regression analyses of predictors for all-cause mortality.

Variables	HR (95% CI)	*p* value
Age (per 1 SD increment)	1.18 (0.99–1.41)	0.07
Chronic kidney disease	1.23 (0.80–1.89)	0.35
Hyperlipidemia	0.83 (0.62–1.12)	0.23
Hyperuricemia	0.95 (0.69–1.30)	0.73
Previous myocardial infarction	1.40 (1.00–1.96)	0.05
Acute coronary syndrome	0.74 (0.47–1.16)	0.19
Acute decompensated heart failure	0.67 (0.45–1.01)	0.06
Systolic blood pressure (per 1 SD increment)	0.90 (0.78–1.04)	0.14
Hemoglobin (per 1 SD increment)	0.81 (0.70–0.94)	0.01
eGFR on admission (per 1 SD increment)	0.98 (0.79–1.22)	0.88
Log NT-proBNP (per 1 SD increment)	1.89 (1.49–2.40)	< 0.001
Log Troponin I (per 1 SD increment)	1.11 (0.95–1.30)	0.18
LVEF (per 1 SD increment)	0.85 (0.72–1.00)	0.05
CAG or PCI before admission	0.90 (0.62–1.32)	0.59
Log RAI (per 1 SD increment)	1.29 (1.12–1.49)	< 0.001

The multivariable model was adjusted for all baseline variables with *p* < 0.05 in univariate analyses. *HR* hazard ratio; *CI* confidence interval; *SD* standard deviation; *eGFR* creatinine-based estimated glomerular filtration rate; *Log NT-proBNP* logarithm N-terminal pro-B-type natriuretic peptide; *Log Troponin I* logarithm Troponin I; *LVEF* left ventricular ejection fraction; *CAG* coronary angiography; *PCI* percutaneous coronary intervention; *Log RAI* logarithm renal angina index.

## Discussion

The main results of this study were as follows: First, the RAI was a significant independent predictor of severe AKI in patients hospitalized in CICUs. Second, the RAI was associated with all-cause mortality along with NT-proBNP. Thus, the RAI is a potent and independent predictor not only of severe AKI but also of adverse outcomes and could improve the risk stratification of patients hospitalized in the CICU.

The utility of the RAI has already been validated in critically ill children^13–15^. The RAI for critically ill children cannot be extrapolated to adult patients because of their differences in comorbidities and risk factors for AKI. Matsuura et al.^3^ proposed a modified RAI for critically ill adult patients and demonstrated an independent association between the RAI and persistent AKI in a cohort of 2 separate prospective studies. However, only 7.6% of those patients had cardiovascular disease. CICUs provide comprehensive critical care for patients with a range of acute cardiovascular illnesses and complex comorbidities^16,17^. These include diabetes mellitus, hypertension, dyslipidemia, CKD and those who use therapeutic devices that are associated with the incidence of AKI^18,19^. Few studies have examined the usefulness of the RAI in CICU settings, and there is a need to confirm its reliability and generalizability in heterogeneous populations before its clinical use can be advocated. Thus, we focused on patients with cardiovascular disease and demonstrated, for the first time to our knowledge, an independent association between the RAI and severe AKI in a large (n = 2,436), heterogeneous cohort of patients treated in CICUs.

We used the previously reported RAI^3^ for patients hospitalized in the CICU. Our data showed that the optimal RAI cutoff value for predicting severe AKI was 4 points, which was lower than that of a previous study (6 points)^3^. The difference in the cutoff value may be attributed to patient characteristics (cardiovascular disease: 7.6%). In the present study, patients who underwent IABP or PCPS had a high risk of severe AKI, suggesting that the addition of mechanical circulatory support to the RAI may further improve the predictive value of severe AKI in patients with cardiovascular disease. The RAI for patients with cardiovascular disease requires further studies in other CICU settings and remains to be validated in larger cohorts.

NT-proBNP was not a significant predictor of severe AKI in this study. Natriuretic peptides are a well-known biomarker of cardiac volume and hemodynamics, but they have been evaluated as AKI biomarkers in patients with heart failure and acute coronary syndrome^20,21^. We previously reported the predictive value of NT-proBNP for AKI^6^. Fiorentino et al. demonstrated that the addition of day-14 plasma B-type natriuretic peptide to the clinical model significantly improved the prediction of renal recovery in critically ill patients^22^. Additional high-quality studies are needed to clarify the usefulness of natriuretic peptides for predicting AKI.

AKI is strongly associated with increased morbidity, mortality, and the long-term loss of kidney function^23–25^. NT-proBNP is a well-established cardiac-specific biomarker for predicting the risk of cardiovascular events and death in patients with cardiovascular disease^26–28^. Our results indicate that the RAI, like NT-proBNP, is a significant independent predictor of 12-month mortality in patients hospitalized in CICUs. Considering that NT-proBNP provides information different from that provided by the RAI, the assessment using hemodynamic stress markers and AKI risk scores may be clinically beneficial. Risk assessment by the RAI and NT-proBNP may stratify 12-month all-cause mortality risk in patients treated in CICUs and identify high-risk patients who will benefit from more aggressive treatments. Each predictor is readily measured, easily accessible, and relatively inexpensive. Therefore, assessment of both NT-proBNP and the RAI score is simple, has a robust discriminative capacity, and may help in risk stratification of patients hospitalized in CICUs.

In the present study, we could not perform multivariate analyses for progression to ESKD because of the small number of events (n = 20). Patients who progressed to ESKD had higher RAI values than those who did not (10 vs. 5 points, *p* = 0.02), suggesting that RAI may be a useful tool for predicting renal outcome.

### Study limitations

Our study had several limitations. First, the retrospective analyses were conducted at a single center. Second, AKI was only defined according to SCr increase because of the inconsistent data recorded and the potential alterations in urine volume induced by medical therapy. This limitation might have led us to neglect part of the renal insult, which might be determined by urine output. Third, it is also worth mentioning that treatments were not randomized in the present study, so it is difficult to evaluate their effects on the primary endpoint. Therefore, we did not evaluate drug treatment using Cox multivariate analysis. Patients with all-cause death used ARB or ACE inhibitors and beta-blockers less frequently than those without. Therefore, differences in medications may have potentially confounded our results. However, when we entered these medications into our Cox multivariate analyses, the RAI was an independent predictor of all-cause death. Consequently, we believe that medication did not significantly affect our results. In this study, 29 patients who died within 24 h were excluded; all patients needed RRT. This may have contributed to the low number of ESKD cases (20 cases).

## Conclusion

The RAI is a potent predictor not only of severe AKI but also of adverse outcomes. It would substantially improve the 12-month risk stratification of patients hospitalized in the CICU.

### Supplementary Information

Supplementary Tables.

## Data Availability

All data generated or analyzed during this study are included in this published article.
