# Supplementary material for: Assessment of the renal angina index in patients hospitalized in a cardiac intensive care unit
Source: Sci Rep. 2024 Jan 2;14:75. doi: 10.1038/s41598-023-51086-0 (PMC10762003; doi:10.1038/s41598-023-51086-0)
Supplement: Supplementary file 1 — Supplementary Tables. [file 41598_2023_51086_MOESM1_ESM.doc]

|  | Severe AKI (-) | Severe AKI (+) | *p*-value |
| --- | --- | --- | --- |
| Number | 2,349 | 87 |  |
| Male | 1,520 (65) | 52 (60) | 0.35 |
| Age (years) | 70.3 ± 13.4 | 72.8 ± 12.0 | 0.09 |
| Hypertension | 1,490 (63) | 65 (75) | 0.03 |
| Diabetes mellitus | 826 (35) | 35 (40) | 0.34 |
| Hyperlipidemia | 1,011 (43) | 38 (44) | 0.91 |
| Hyperuricemia | 543 (23) | 26 (30) | 0.15 |
| Previous myocardial infarction | 351 (15) | 15 (17) | 0.56 |
| Chronic kidney disease | 1,044 (44) | 64 (74) | <0.001 |
| Acute coronary syndrome | 1,051 (45) | 32 (37) | 0.14 |
| Acute decompensated heart failure | 829 (35) | 42 (48) | 0.01 |
| Systolic blood pressure (mmHg) | 137 ± 27 | 139 ± 31 | 0.36 |
| Heart rate (bpm) | 88 ± 30 | 92 ± 31 | 0.27 |
| Hemoglobin (g/dL) | 12.4 ± 2.2 | 11.5 ± 2.3 | <0.001 |
| Serum creatinine at admission (mg/dL) | 0.96 ± 0.43 | 1.25 ± 0.65 | <0.001 |
| Serum creatinine after 24 hours (mg/dL) | 1.00 ± 0.47 | 1.68 ± 0.97 | <0.001 |
| eGFR at admission (mL/min/1.73 m^2^) | 81.4 ± 24.6 | 65.7 ± 26.9 | <0.001 |
| NT-proBNP (pg/mL) | 1,433  (282–4,817) | 3,843  (1,070–12,246) | <0.001 |
| Troponin I (ng/mL) | 0.17 (0.03–2.54) | 0.24 (0.04–3.26) | 0.001 |
| Ventilation at enrollment | 419 (18) | 40 (46) | <0.001 |
| Vasopressor at enrollment | 433 (18) | 50 (57) | <0.001 |
| IABP or PCPS at enrollment | 152 (6) | 22 (25) | <0.001 |
| CAG or PCI before admission | 768 (33) | 24 (28) | 0.31 |
| RAI | 3 (1–5) | 8 (5–24) | <0.001 |
| LVEF (%) | 47 ± 14 | 43 ± 14 | 0.03 |

Supplementary Table S1. Baseline characteristics according to severe AKI. Data are presented as number (%), mean ± standard deviation, or median (interquartile range). AKI, acute kidney injury; bpm, beats per minute; eGFR, creatinine-based estimated glomerular filtration rate; NT-proBNP, N-terminal pro-B-type natriuretic peptide; IABP, intra-aortic balloon pumping; PCPS, percutaneous cardio pulmonary support; CAG, coronary angiography; PCI, percutaneous coronary intervention; RAI, renal angina index; LVEF, left ventricular ejection fraction.

| Variables | AUC (95% CI) | *p*-value |
| --- | --- | --- |
| Male | 0.52 (0.47–0.58) | 0.35 |
| Age | 0.55 (0.49–0.61) | 0.09 |
| Hypertension | 0.56 (0.51–0.60) | 0.03 |
| Diabetes mellitus | 0.53 (0.47–0.58) | 0.33 |
| Hyperlipidemia | 0.50 (0.45–0.56) | 0.91 |
| Hyperuricemia | 0.53 (0.48–0.58) | 0.15 |
| Previous myocardial infarction | 0.51 (0.47–0.55) | 0.56 |
| Chronic kidney disease | 0.65 (0.60–0.69) | <0.001 |
| Acute coronary syndrome | 0.54 (0.49–0.59) | 0.14 |
| Acute decompensated heart failure | 0.56 (0.51–0.62) | 0.01 |
| Systolic blood pressure | 0.53 (0.46–0.59) | 0.36 |
| Heart rate | 0.53 (0.47–0.60) | 0.27 |
| Hemoglobin | 0.62 (0.56–0.68) | <0.001 |
| Serum creatinine at admission | 0.63 (0.57–0.70) | <0.001 |
| Serum creatinine after 24 hours | 0.75 (0.69–0.81) | <0.001 |
| eGFR at admission | 0.67 (0.61–0.73) | <0.001 |
| Log NT-proBNP | 0.66 (0.60–0.71) | <0.001 |
| Log Troponin I | 0.53 (0.47–0.59) | 0.25 |
| Ventilation at enrollment | 0.64 (0.59–0.69) | <0.001 |
| Vasopressor at enrollment | 0.70 (0.64–0.75) | <0.001 |
| IABP or PCPS at enrollment | 0.59 (0.55–0.64) | <0.001 |
| CAG or PCI before admission | 0.53 (0.48–0.57) | 0.32 |
| Log RAI | 0.80 (0.75–0.85) | <0.001 |
| LVEF | 0.58 (0.52–0.64) | 0.03 |

Supplementary Table S2. Univariate logistic analyses of AUC for severe AKI. All variables have *p*-value<0.05 in univariate analyses. AUC, area under the receiver operating characteristic curve; AKI, acute kidney injury; CI, confidence interval; SD, standard deviation; eGFR, creatinine-based estimated glomerular filtration rate; Log NT-proBNP, logarithm N-terminal pro-B-type natriuretic peptide; Log Troponin I, logarithm Troponin I; IABP, intra-aortic balloon pumping; PCPS, percutaneous cardio pulmonary support; CAG, coronary angiography; PCI, percutaneous coronary intervention; Log RAI, logarithm renal angina index, LVEF, left ventricular ejection fraction.

| Variables | AUC (95% CI) | *p*-value |
| --- | --- | --- |
| Male | 0.50 (0.47–0.54) | 0.82 |
| Age | 0.63 (0.59–0.66) | <0.001 |
| Hypertension | 0.51 (0.48–0.55) | 0.54 |
| Diabetes mellitus | 0.51 (0.47–0.54) | 0.68 |
| Hyperlipidemia | 0.54 (0.51–0.57) | 0.03 |
| Hyperuricemia | 0.54 (0.51–0.57) | 0.01 |
| Previous myocardial infarction | 0.55 (0.52–0.58) | <0.001 |
| Chronic kidney disease | 0.64 (0.61–0.67) | <0.001 |
| Acute coronary syndrome | 0.60 (0.57–0.63) | <0.001 |
| Acute decompensated heart failure | 0.61 (0.57–0.64) | <0.001 |
| Systolic blood pressure | 0.55 (0.51–0.58) | 0.03 |
| Heart rate | 0.55 (0.51–0.59) | 0.09 |
| Hemoglobin | 0.66 (0.62–0.70) | <0.001 |
| Serum creatinine at admission | 0.67 (0.63–0.71) | <0.001 |
| Serum creatinine after 24 hours | 0.68 (0.64–0.72) | <0.001 |
| eGFR at admission | 0.69 (0.66–0.73) | <0.001 |
| Log NT-proBNP | 0.75 (0.72–0.78) | <0.001 |
| Log Troponin I | 0.55 (0.51–0.59) | 0.03 |
| Ventilation at enrollment | 0.61 (0.57–0.64) | <0.001 |
| Vasopressor at enrollment | 0.64 (0.61–0.68) | <0.001 |
| IABP or PCPS at enrollment | 0.51 (0.49–0.53) | 0.16 |
| CAG or PCI before admission | 0.56 (0.53–0.59) | <0.001 |
| Log RAI | 0.67 (0.63–0.71) | <0.001 |
| LVEF | 0.65 (0.62–0.69) | <0.001 |

Supplementary Table S3. Univariate logistic analyses of AUC for all-cause mortality. All variables have *p*-value<0.05 in univariate analyses. AUC, area under the receiver operating characteristic curve; CI, confidence interval; SD, standard deviation; eGFR, creatinine-based estimated glomerular filtration rate; Log NT-proBNP, logarithm N-terminal pro-B-type natriuretic peptide; Log Troponin I, logarithm Troponin I; CAG, coronary angiography; PCI, percutaneous coronary intervention; CAG, coronary angiography; PCI, percutaneous coronary intervention; Log RAI, logarithm renal angina index, LVEF, left ventricular ejection fraction.
